# Competence and training needs in cancer pain management among nurses: a cross-sectional study

**DOI:** 10.3389/fmed.2025.1594859

**Published:** 2025-08-22

**Authors:** Ting Dong, Lingcao Ma, Xiaolin Song, Sisi Wang, Hanqing Dai, Kunhua Hou

**Affiliations:** ^1^Department of Otolaryngology Head and Neck Surgery, Henan Provincial People’s Hospital, People’s Hospital of Zhengzhou University, Zhengzhou, Henan, China; ^2^Department of Neurology, Henan Provincial People’s Hospital, People’s Hospital of Zhengzhou University, Zhengzhou, Henan, China; ^3^Department of Dermatology, Henan Provincial People’s Hospital, People’s Hospital of Zhengzhou University, Zhengzhou, Henan, China

**Keywords:** nurse, cancer pain, competence, empathy, emotional intelligence, educational need

## Abstract

**Background:**

Effective cancer pain management remains a critical global healthcare priority, however, significant disparities in nursing competence persist across healthcare systems. Although previous research has identified deficiencies in pain management practices, comprehensive evaluations of nurses’ competence specifically in cancer pain management and their associated training needs are limited. This study contributes to the existing body of knowledge by assessing nurses’ competency levels, identifying key modifiable factors that influence performance, and examining both the current status of training and unmet educational needs.

**Methods:**

This cross-sectional study included a sample of 202 nurses. Cancer pain management competence was assessed using the Nurses’ Cancer Pain Management Competency Scale. Group comparisons were conducted using *t* test and analysis of variance. Associated factors were analyzed through Pearson’s correlation analysis and multivariate linear regression.

**Results:**

Nurses demonstrated a moderate level of competence on the Nurses’ Cancer Pain Management Competency Scale. Multiple linear regression analysis revealed that age, pain or cancer pain training, frequency of cancer pain care, and emotional intelligence were significant predictors of cancer pain management competence, collectively explaining 50.1% of the variance in scores (*F* = 23.455, *p* < 0.01). While 89.6% of nurses reported a need for cancer pain management training, only 47.53% have received such training in the past 3 months. E-learning was the most preferred educational format, followed by lectures, hands-on practical training, and simulation-based learning.

**Conclusion:**

This study highlights that nurses’ competence in cancer pain management is significantly influenced by age, pain or cancer pain training, frequency of cancer pain care, and emotional intelligence. Despite a high demand for education, recent participation in training remains suboptimal. The preference for e-learning indicates a growing inclination toward flexible and accessible training approaches. Addressing these gaps in competence and training is crucial for improving the quality of cancer pain management in clinical practice.

## 1 Introduction

Cancer pain remains a critical global health challenge, significantly impacting patients’ survival rates, treatment adherence, and quality of life (1, 2). Despite its profound implications, cancer pain management remains inadequate worldwide, with particularly pronounced disparities observed in developing countries (3–7). This study aims to comprehensively assess nurses’ cancer pain management competencies, explore the impact of emotional intelligence, and provide practical data on nurses’ preferred learning styles.

Nurses play a central role in cancer pain management through continuous assessment, intervention, and evaluation (8). Nurse-led programs have demonstrated effectiveness in enhancing pain control and clinical outcomes (9). However, nurses across healthcare systems consistently report substantial barriers of effective cancer pain management (10, 11). Regional disparities exist, with studies indicating that nurses in Asian countries tend to exhibit lower levels of knowledge compared to their counterparts in Western countries (12–14). In China, studies highlight specific deficits in acute pain management and individualized care planning, with correct response rates on competency surveys below 60% (15, 16). Preliminary studies have reported that nurses’ comprehensive competency in cancer pain management remains suboptimal. However, the factors influencing cancer pain management competency have not been systematically reported. Tailored training programs are needed to enhance nurses’ abilities in delivering optimal cancer pain management (11, 17, 18). Assessing nurses’ identified educational needs provides a practical foundation for developing training plans (19). Although a few studies have briefly surveyed the educational needs related to pain management among nurses, they revealed that nurses strongly needed training, coupled with low recent attendance in cancer pain training (8, 20). Furthermore, there is a notable lack of comprehensive assessment of nurses’ access to learning resources and their specific educational needs. One study found that nurses’ emotional intelligence positively influences their awareness of pain management (21), but its influence on their cancer pain management competency remains unreported. Therefore, although previous research has addressed aspects of pain management-related knowledge and attitudes, comprehensive evaluations of nurses’ cancer pain management competency, along with an assessment of their training needs and resources, remain limited.

Therefore, the specific objectives of this study were to (a) assess the competence level of nurses in cancer pain management; (b) identify key influencing factors, such as demographics, training history, and psychosocial traits (e.g., empathy, emotional intelligence); and (c) evaluate educational needs and preferences for training resources.

The study was guided by the following three hypotheses:

*Hypothesis 1:* There are differences in cancer pain management competence among nurses by demographic and training history characteristic variables.

*Hypothesis 2:* Nurses’ psychosocial traits particularly empathy, emotional intelligence, are associated cancer pain management competence.

*Hypothesis 3:* Nurses have high training needs and preferences for training methods.

## 2 Methods

### 2.1 Study design and participants

The cross-sectional study was conducted from August 27, 2024 to September 5, 2024, using a convenience sampling method. Data were collected from three tertiary hospitals in Zhengzhou, China. Nurses were eligible for participating into this study if they met the following criteria: (a) held a valid registered nurse certification, (b) had worked at the hospitals for more than 1 year, and (c) provided informed consent and volunteered to participate in this study. Nurses not working at the selected hospitals were excluded from the study.

### 2.2 Sample size calculation

According to the sample size calculation principle for cross-sectional studies, the required sample size should be 5–10 times the total number of items or dimensions in the questionnaire (22). To account for potential non-responses or data loss, the calculated sample size was increased by an additional 10%. The questionnaire consisted of 24 items and dimensions, resulting in an estimated sample size ranged of 132–264 cases. A total of 210 questionnaires were distributed, however, 8 incomplete response questionnaires (3.81%) were excluded, yielding an effective response rate of 96.19%.

### 2.3 Data collection

The research team contacted the head nurses of those three hospitals to explain the purpose and content of the study and obtained their approval. A trained investigator then distributed the questionnaires to the nurses who consented to participate and collected them on the spot. The investigator reviewed the retrieved questionnaires, and for items that were left blank, the research subjects were requested to provide supplementary data. Questionnaires with a missing response rate exceeding 5%—due to either research subjects withdrawing from the study midway or refusing to supplement the blank items—were excluded from the analysis. Participants were informed about the purpose and significance of the study, the voluntary nature of the survey, and the confidentiality of their responses before the questionnaire was distributed. In addition, as an incentive to participate in the study, nurses who completed the survey were given a small gift, such as a hair clip or a keychain. After data collection and organization, the results were shared with nursing administrators, and the data analysis was conducted to evaluate the current state of nurses’ competence in cancer pain management.

### 2.4 Instruments

#### 2.4.1 Demographic characteristics questionnaire

Demographic data were collected using a questionnaire designed by the research team. Variables included age, work unit, education level, marital status, professional title, and work experience.

#### 2.4.2 Nurses’ cancer pain management competency

Nurses’ cancer pain management competency was measured using the Nurses’ Cancer Pain Management Competency Scale (NCPMCS), developed by Hu and Roh (8). The scale comprises 14 items across four dimensions: the context of pain management, pain assessment and measurement, management of pain, and multidimensional nature of pain. Items are rated on a 4-point Likert scale ranging from 1 (poor) to 4 (excellent). Higher scores demonstrate greater self-assessment competency. The NCPMCS has indicated excellent reliability (Cronbach’s *α* = 0.890), and in this study, Cronbach’s α was 0.948.

#### 2.4.3 The Jefferson scale of empathy

A 20-item instrument developed by Hojat et al. (23). The scale comprises three factors as follows: perspective-taking, compassionate care, and “walking in patient’s shoes.” Responses are measured on a 7-point Likert scale ranging from 1 (strongly disagree) to 7 (strongly agree), with higher scores associated with stronger empathy. The Cronbach’s *α* for this scale is 0.750.

#### 2.4.4 Emotional intelligence scale

Emotional intelligence was evaluated using the Wong and Law Emotional Intelligence Scale, designed by Wong and Law (24). It contains 16 items and four dimensions (self-emotional appraisal, others’ emotional appraisal, regulation of emotion, and use of emotion). Each item is rated on a 7-point Likert scale ranging from 1 (strongly disagree) to 7 (strongly agree). Cronbach’s *α* for this scale is 0.830.

#### 2.4.5 Nurses’ cancer pain management educational resources and needs

Nurses’ Pain Management Educational Resources and Needs related to cancer pain management were assessed using a questionnaire developed by the researchers. During the questionnaire design process, existing literature (8, 20) were consulted. Furthermore, three nursing experts with specialized knowledge in clinical practice and education were invited to provide guidance on the questionnaire content. It included seven items addressing nurses’ current practices and training needs as follows: (1) when did you last receive pain management training? (past 1 month, past 3 months, more than 1 year, or never). (2) When did you last receive cancer pain management training? (past 1 month, past 3 months, more than 1 year, or never). (3) How frequently do you engage in cancer pain care? (frequently, generally, sometimes, or never). (4) What is your primary source of knowledge on cancer pain management? (clinical practice, continuing education, formal education, personal experience, e-learning, or literature review). (5) Do you think cancer pain management training is necessary? (yes, no, or indifferent). (6) Do you personally feel the need for cancer pain management training? (very necessary, necessary, or unnecessary). (7) What is your preferred educational training modality? (lectures, e-learning, simulation-based learning, or hands-on practical training).

### 2.5 Statistical analysis

Data were analyzed using IBM SPSS Statistics version 27.0 (IBM Corp., Armonk, NY, USA). The software expressed categorical variables data as frequencies and percentages, while continuous variables was reported as arithmetic means and standard deviations (SDs). The normality of continuous variables was assessed using Q-Q plots, which indicated an approximate normal distribution. Group differences were examined using Student’s *t*-test and one-way analysis of variance (ANOVA). Pearson’s correlation and multiple linear regression models were conducted to identify significant factors associated with Nurses’ Cancer Pain Management Competency Scale (NCPMCS) scores. A *p* < 0.05 was considered statistically significant. All statistical tests were two-tailed. Applying multiple imputation to handle missing data in statistical analysis.

### 2.6 Ethics approval

This study was approved by the Ethics Committee of Henan Provincial People’s Hospital [(2022) Lun review no. (24)]. The study followed the Strengthening the Reporting of Observational Studies in Epidemiology guidelines. Informed consent was obtained from all participants.

## 3 Results

### 3.1 Demographic characteristics of nurses

The average age of the participating nurses was 35 years. Nearly half were employed in oncology ward (49.51%). The majority held a bachelor’s degree (91.09%), were married (84.65%), and held the title of nurse-in-charge (66.34%) (Table 1).

**Table 1:** Demographic characteristics of nurses (*N* = 202).

Variable	*N* (%)	NCPMCS mean	NCPMCS SD	t/F	*p*-value	Cohens’d/Partial *η*^2^
Work unit						
Oncology ward	100 (49.51)	44.02	7.43	14.724^b^	**<0.001**	0.182
Intensive care unit	18 (8.91)	41.61	10.79			
Medical ward	32 (15.84)	39.38	8.50			
Surgical ward	52 (25.74)	35.48	6.11			
Education level						
College and below	4 (1.98)	41.25	8.62	0.030^b^	0.971	0.001
Bachelor’s degree	184 (91.09)	40.83	8.34			
Master’s degree and above	14 (6.93)	41.36	9.63			
Marital status						
Unmarried	31 (15.35)	40.42	9.41	−0.325^a^	0.745	0.063
Married	171 (84.65)	40.95	8.23			
Professional title						
Nurse practitioner and below 46 (22.77)	39.80	9.01	0.628^b^	0.535	0.006	
Nurse-in-charge	134 (66.34)	41.04	8.31			
Associate Chief Nurse	22 (10.89)	42.09	7.71			
Time of receiving pain management training						
Past 1 month	58 (28.71)	46.02	6.83	22.836^b^	**<0.001**	0.257
Past 3 months	59 (29.21)	41.54	8.19			
More than 1 year	67 (33.17)	38.42	7.24			
Never	18 (8.91)	31.22	5.49			
Time of receiving cancer pain management training						
Past 1 month	43 (21.29)	47.51	5.72	31.839^b^	**<0.001**	0.325
Past 3 months	53 (26.24)	43.70	7.83			
More than 1 year	56 (27.72)	38.82	7.48			
Never	50 (24.75)	34.46	6.26			
Frequency of cancer pain care						
Frequently	70 (34.65)	46.04	6.37	22.603^b^	**<0.001**	0.255
Generally	61 (30.20)	40.28	8.37			
Sometimes	47 (23.27)	37.55	7.20			
Never	24 (11.88)	33.79	7.10			

^a^*t*-test, ^b^ANOVA, NCPMCS, Nurses’ Cancer Pain Management Competency Scale. Boldface indicates variables with statistical significance.

### 3.2 NCPMCS scores

The total score of NCPMCS was 40.87 (SD = 8.40), with an average item score of 2.92 (SD = 0.60). Among the four dimensions of NCPMCS, pain assessment and measurement scored the highest [2.99 (SD = 0.66)], followed by clinical conditions [2.94 (SD = 0.64)], pain management [2.88 (SD = 0.76)], and multidimensional nature of pain [2.71 (SD = 0.66)] (Table 2).

**Table 2:** Nurses’ cancer pain management competency scale score and scores of the four dimensions (*N* = 202).

Variable	Number of items	Mean (SD) of total scores	Mean (SD) of item scores
Total score of NCPMCS	14	40.87 (8.40)	2.92 (0.60)
Clinical conditions	5	14.74 (3.18)	2.94 (0.64)
Pain assessment and measurement	5	14.96 (3.30)	2.99 (0.66)
Management of pain	2	5.76 (1.52)	2.88 (0.76)
Multidimensional nature of pain	2	5.42 (1.32)	2.71 (0.66)

NCPMCS, Nurses’ cancer pain management competency scale.

### 3.3 Cancer pain management competency scores by demographic characteristics

Univariate analyses showed significant differences in NCPMCS scores by work unit, pain or cancer pain management training, and frequency of cancer pain care (*p* < 0.01). Nurses who had recently received pain or cancer pain management training and those who regularly cared for patients with cancer pain had higher competence (Table 1).

Pearson’s correlation coefficient showed a positive correlation between NCPMCS and age (*r* = 0.281, *p* < 0.01), work experience (*r* = 0.176, *p* < 0.05), empathy scale (*r* = 0.139, *p* < 0.05), and emotional intelligence scale (*r* = 0.332, *p* < 0.01) (Table 3).

**Table 3:** Pearson’s correlation coefficient between NCPMCS and possible influencing factors.

Variables	Mean of variables	SD of variables	Correlation coefficient of NCPMCS
Age (year)	34.574	6.200	0.281^a^
Work experience (year)	12.802	6.696	0.176^b^
Empathy scale	116.140	16.431	0.139^b^
Compassionate care	49.965	3.486	0.703^a^
Perspective taking	63.352	3.922	0.783^a^
Walking in patient’s shoes	13.059	1.040	0.592^a^
Emotional intelligence scale	84.500	15.030	0.332^a^
Self-emotional appraisal	24.198	2.294	0.356^a^
Others’ emotional appraisal	23.183	2.991	0.285^a^
Use of emotion	23.946	2.478	0.263^a^
Regulation of emotion	23.010	3.004	0.237^a^

SD, standard deviation; NCPMCS, Nurses’ Cancer Pain Management Competency Scale. ^a^*p* < 0.01, ^b^*p* < 0.05.

### 3.4 Multiple linear regression analysis

Multiple linear regression was performed with NCPMCS scores as the dependent variable and variables found to be statistically significant in univariate analyses as independent predicators: work unit, age, empathy scale, emotional intelligence scale, pain or cancer pain management training, and frequency of cancer pain care. Emotional intelligence, age, oncology ward, pain or cancer pain management training, and frequency of cancer pain care explained 50.1% of the variance in NCPMCS scores (*F* = 23.455, *p* < 0.01) (Table 4).

**Table 4:** Regression analysis of NCPMCS and possible influencing factors.

Variable	Coefficient (*β*)	*β’*	SE	*t*	*p-*value	95% confidence interval		Tolerance	VIF
						Lower	Upper		
b	33.989		4.858	6.996	**<0.01**	24.407	43.571		
Empathy	0.034	0.066	0.027	1.240	0.217	−0.020	0.087	0.880	1.136
Emotional intelligence	0.106	0.189	0.030	3.552	**<0.01**	0.047	0.165	0.873	1.146
Age	0.172	0.127	0.070	2.458	**0.015**	0.034	0.310	0.929	1.077
Pain management training	−1.543	−0.177	0.578	−2.671	**0.008**	−2.683	−0.404	0.563	1.776
Cancer pain management training	−1.828	−0.236	0.538	−3.395	**<0.01**	−2.889	−0.766	0.514	1.944
Frequency of cancer pain care	−1.935	−0.235	0.474	−4.080	**<0.01**	−2.870	−0.999	0.745	1.343
Work unit									
Oncology ward	3.602	0.215	1.108	3.250	**0.001**	1.416	5.788	0.567	1.765
Intensive care unit	2.046	0.070	1.684	1.215	0.226	−1.276	5.368	0.756	1.323
Medical ward	1.747	0.076	1.376	1.270	0.206	−0.967	4.461	0.689	1.451

F = 23.455, *p* < 0.001; R = 0.724, R^2^ = 0.524, adjusted R^2^ = 0.501; NCPMCS, Nurses’ Cancer Pain Management Competency Scale. Boldface indicates variables with statistical significance.

### 3.5 Nurses’ cancer pain management educational resources or needs

Among the 202 nurses, 131 (64.85%) reported regularly providing cared for patients with cancer pain; however, only 96 (47.53%) had received cancer pain management training within the past 3 months (Table 1). More than one-half of the nurses (*n* = 116, 57.4%) learned about cancer pain management knowledge through two or more modalities. The main modalities were clinical practice (85.15%), continued education (77.72%), and literature review (64.85%).

Furthermore, 92.08% of nurses believed that cancer pain management training should be implemented, and 89.6% of nurses needed for such training. A total of 144 nurses (71.3%) indicated a preference for various educational training modalities (> 2 methods); the most preferred educational modality was e-learning (79.7%), followed by lectures (60.89%), hands-on practical training (59.41%), and simulation-based learning (49.5%) (Table 5).

**Table 5:** Nurses’ cancer pain management educational resources and needs (*N* = 202).

Category	*N* (%)
Sources of knowledge about cancer pain management	
Clinical practice	172 (85.15)
Continuing education	157 (77.72)
Formal education	42 (20.79)
Personal experience	64 (31.68)
E-learning	80 (39.60)
Literature review	131 (64.85)
Do you need cancer pain management training?	
Yes	181 (89.60)
No	21 (10.40)
Need for cancer pain management education	
Very necessary	106 (52.48)
Necessary	87 (43.07)
Unnecessary	9 (4.46)
What is your preferred educational modality?	
Lectures	123 (60.89)
E-learning	161 (79.70)
Simulation-based learning	100 (49.50)
Hands-on practical training	100 (49.50)

## 4 Discussion

Effective pain management can control pain in 70–90% of the patients with cancer, which illustrates that the pain management significantly improving their physical, psychological, and spiritual wellbeing (25). A study found no significant correlation between nurses’ knowledge of cancer pain and cancer pain management practice competence (20). This highlights the importance of the competence in cancer pain management. The NCPMCS was developed based on the core pain management competencies identified by Fishman et al. (26). It offers a comprehensive framework to assess nurses’ pain management competency and serve both as a needs assessment instrument for nurses and as an assessment instrument to evaluate learning outcomes after their training. In this study, the NCPMCS was applied to preliminarily assess nurses’ cancer pain management competency, offering a foundation for subsequent in-depth research.

According to the prior research (15, 27, 28), nurse from developing countries often score lower on cancer pain management knowledge and attitudes compared to those in developed countries. Consistent with those in other studies (8, 25), NCPMCS scores in our study population were moderate (40.87 ± 8.40 points on a 14–56 point scale). In contrast to the findings of Hu and Roh (8), our study revealed that the highest subscale score was in pain assessment and measurement (2.99 ± 0.66), while the lowest was in understanding the multidimensional nature of pain (2.71 ± 0.66). Similarly, Yu reported that nurses’ knowledge of pain assessment and measurement was high in China (16). The moderate competency scores highlight a critical gap in nurses’ ability to manage cancer pain effectively, particularly in understanding the multidimensional nature of pain. This underscores the need for targeted educational programs that address these deficits, with a focus on holistic pain management approaches.

We found that age was an influential factor in cancer pain management competence. This aligns with prior two studies from China and Korea, both of which reported positive associations between age and knowledge of cancer pain management (16, 29). Pain is an unpleasant sensory and emotional experience associated with tissue damage or potential tissue damage (30). Emotional intelligence enables individuals to monitor the emotions of self and others to guide their thoughts and behaviors by identifying and using this information (31). Issa et al. (21) reported nurses’ emotional intelligence positively influences their awareness of pain management. Consistently, our study showed that the higher the emotional intelligence was associated with better cancer pain management competence. This suggests that integrating emotional intelligence training into professional development programs may enhance nurses’ ability to monitor and regulate patients’ emotions, thereby improving pain management outcomes. Furthermore, younger nurses should be prioritized in educational initiatives to bridge gaps in clinical competence.

Previous studies have shown that nurses who received formal training in pain management possess higher levels of pain management knowledge than those without training (16, 29, 32). The results in this study were aligned with this fact and demonstrated that pain or cancer pain management training impacted NCPMCS scores. Nurses without such training exhibited reduced competence, which indicate a need for training programs (10, 15, 33).

In addition, our study showed that nurses who frequency provided cancer pain care and those working in oncology ward had significantly higher competence level. This may be attributed to more frequent care of cancer patients with pain and learning reinforcement. These findings suggest that expanding relevant clinical training opportunities and encouraging non-oncology nurses to engage in cancer pain care may help increase competency levels. Correspondingly, non-oncology nurses should be actively encouraged to participate in cancer pain management nursing practice and supported through target educational training programs.

Notably, the regression model in this study exhibited an unexplained variance rate of 49.9%, which indicates that the independent variable has certain limitations in explaining the dependent variable. The unexplained variance may be attributable to other unmeasured factors, such as training methods and individual difference in nurses’ learning abilities. Future research is recommended to further explore other influencing factors of nurses’ cancer pain management competence to provide more comprehensive guidance for the development of training programs.

In our study, 95.55% of the nurses believed that cancer pain management training is necessary, and 89.6% of nurses reported a personal needed for training. These findings are consistent with Sook’s study, in which 93.3% of the nurses believed that pain management education is necessary (20). Approximately 75.25% of the participating nurses were trained in cancer pain management, aligning with Hu and Roh’s (8) reported rate of 76.5%. The high demand for training, coupled with low recent participation, indicates a systemic issue in providing continuous professional development. A majority of 172 (85.1%) nurses obtained cancer pain management knowledge through more than two modes. The top three were clinical practice, continued education, and literature review. Regarding preferred training modalities, 144 (71.3%) nurses expressed interest in training through two or more formats. E-learning was the most preferred method, followed by lectures, hands-on practical training, and simulation-based training. The preference for e-learning suggests that flexible and accessible training modalities could enhance participation and engagement, particularly for nurses with demanding schedules.

## 5 Limitations

This study has several limitations. The study participants were not selected through stratified random sampling and may not be representative of the entire nursing population. The study was conducted at three hospitals with a relatively small sample size, which may limit the generalizability of the findings to other institutions or regions. Also, the cross-sectional design of the study precludes the determination of causality. Longitudinal research is needed to examine how relevant factors evolve over time and how targeted interventions may affect competence. Furthermore, the “Nurses’ Educational Resources and Needs” questionnaire used in this study lacks psychometric validation, which may affect the reliability of the related findings.

However, this study provides valuable insights for future research. Subsequent studies could consider conducting large-sample longitudinal research to further explore the levels of nurses’ cancer pain management competency and their influencing factors. In-depth exploration of nurses’ training needs and available educational resources is also warranted. Furthermore, effective training programs aimed at developing and sustaining cancer pain management competency among nurses should be implemented. It is recommended to incorporate emotional intelligence training into these programs with a particular emphasis on self-emotional assessment, empathy, and emotion regulation. Training methods should be tailored to align with nurses’ learning preferences, schedules, and individual learning needs.

## 6 Conclusion

This study investigated nurses’ cancer pain management competence, the influencing factors, and the current status and needs of training. The results illustrate that nurses’ competence in cancer pain management remains at a moderate level, with considerable potential for improvement. Factors such as age, emotional intelligence, prior pain or cancer pain management training, and the frequency of cancer pain care were significantly associated with nurses’ competency levels. The study revealed a strong demand for training was identified, with e-learning emerging as the most preferred educational modality among nurses.

## Data Availability

The raw data supporting the conclusions of this article will be made available by the authors, without undue reservation.
